# Negative symptom dimensions and social functioning in Chinese patients with schizophrenia

**DOI:** 10.3389/fpsyt.2022.1033166

**Published:** 2022-12-06

**Authors:** Tianqi Gao, Zetao Huang, Bingjie Huang, Tianhang Zhou, Chuan Shi, Xin Yu, Chengcheng Pu

**Affiliations:** ^1^Peking University Sixth Hospital, Beijing, China; ^2^Peking University Institute of Mental Health, Beijing, China; ^3^National Health Commission Key Laboratory of Mental Health, Peking University, Beijing, China; ^4^National Clinical Research Center for Mental Disorders, Peking University Sixth Hospital, Beijing, China

**Keywords:** schizophrenia, social functioning, negative symptoms, dimensions, motivation

## Abstract

**Objective:**

Negative symptoms can seriously affect social functioning in patients with schizophrenia. However, the role of various components of negative symptoms in social functioning remains unclear. This study aimed to explore the associations among three different dimensions of negative symptoms (i.e., communication, emotion, and motivation) and social functioning to identify potential therapeutic targets.

**Methods:**

This cross-sectional study enrolled 202 Chinese participants with schizophrenia. Negative symptoms were evaluated using the Negative Symptom Assessment (NSA). Social functioning was represented by the Personal and Social Performance Scale (PSP) total score and employment status. Correlation analysis was conducted to clarify the relationship between negative symptoms and the PSP total score. Regression analysis was performed to explore the determinants of the PSP total score and employment status, considering negative symptoms and possible confounders, such as demographic features, positive symptoms, cognitive symptoms, depressive symptoms, and extrapyramidal side effects.

**Results:**

The PSP total score was correlated with all three dimensions of negative symptoms (i.e., emotion, motivation, and communication; *r*s = –0.509, –0.662, and –0.657, respectively). Motivation, instead of emotion or communication, predicted both low PSP total scores and unemployment.

**Conclusion:**

Social functioning in patients with schizophrenia was significantly related to motivation. Further studies should focus on motivation and consider it as a therapeutic target to improve patients’ social functioning.

## Introduction

Patients with schizophrenia generally show impaired social functioning, and they find understanding social contexts, interpersonal communication, and maintaining relationships particularly challenging (1). Impairment in social functioning has a significant negative impact on patients’ lives in both occupation/study and daily life. Therefore, improving social functioning is considered an essential therapeutic objective for patients with schizophrenia (2).

Social functional impairment is strongly associated with negative symptoms and cognitive deficits (1, 3, 4). Negative symptoms play a critical and central role on impacting social performance by directly influencing and mediating cognitive symptoms (5-7). A previous study demonstrated that social functioning was predicted by even a lower level of negative symptoms, which were defined as moderate impairments in two or fewer out of the overall seven sub-domains (8). However, current treatments for negative symptoms provide inadequate relief (9), and most patients experience persistent functional impairments (10).

Although a correlation between negative symptoms and social functioning has been confirmed, the role of various components of negative symptoms remains controversial (11, 12). Clinically, negative symptoms are divided into five domains: anhedonia (inability to experience pleasure), asociality (decreased value of social contacts), avolition (decreased motivation), blunted affect (decrease in non-verbal emotional expression), and alogia (decrease in the amount of speech) (5, 6, 12). Several studies have argued that motivational deficits play an important role in mediating cognitive impairment to induce adverse social functioning (12, 13). Moreover, it has been shown that alogia and avolition predict real-world functioning (14, 15). Another similar study demonstrated that asociality, rather than avolition or alogia, was highly related to social functioning (16). Regarding vocational status as a milestone of social functioning, several studies have shown that avolition, anhedonia, asociality, and alogia all contribute to unemployment (14, 17, 18). Fulford et al. directly explored the association between motivation and functioning and found that motivation is related to current social functioning but not to vocational status (13). However, the relationships among the various domains of negative symptoms and social functioning and employment remain unclear.

Another reason for the inadequate efficacy of treatments is the uncertainty of the mechanisms underlying the structure of negative symptoms, which hinders the identification of potential therapeutic targets (9, 19). Kring and Barch argued that a two-factor structure (i.e., motivation/pleasure and expression) is more conducive to revealing the nature of negative symptoms and finding potential therapeutic targets than the original five-domain structure (10). Okada et al., who support the five-domain structure, suggested that the two-factor model fails to describe the complexity and specificity of the association with functional outcomes (20). Notably, the variety of numbers of factors used to measure negative symptoms are affected by not only the symptomology but the various psychometric property of local population. Manifestation of psychotic symptoms, patients’ performances and physicians’ evaluations may vary from different regions (21–23), where different factor models of the same measurement were generated. For instance, the original five-factor model of the Negative Symptoms Assessment (NSA) was developed by a Western group in the 1980s. Popp et al. and Rekhi et al. used confirmatory factor analyses and proposed three-factor and four-factor structures respectively, based on the various study populations (21, 22). Huang et al. described that negative symptoms vary in structure depending on the demographic and clinical characteristics of the samples and proposed a new three-factor model based on Chinese participants: motivation, expression, and emotion (21).

Therefore, this study aimed to apply an appropriate model of negative symptoms and comprehensively evaluate the relationship between the dimensions of negative symptoms and social functioning to deepen our understanding of negative symptoms and offer potential targets for improving social functioning. Given the central role of motivation in functional outcomes, we hypothesized that motivation would be related to patients’ social functioning and employment status, and the degree of impairment in motivation would be associated with poor social functioning and unemployment.

## Materials and methods

### Subjects

The study was conducted at Peking University Sixth Hospital (PKU6H), Beijing, China, from January 2016 to January 2020.

Participants were screened according to the following inclusion criteria: men and women aged between 16 and 60 years; inpatients or outpatients at PKU6H; and participants who were diagnosed with schizophrenia according to the diagnostic criteria of the Diagnostic and Statistical Manual of Mental Disorders IV (DSM-IV) (24).

Participants were ineligible if they met any of the following exclusion criteria: comorbid diagnosis of another axis I disease according to the DSM-IV (24); severe or unstable medical conditions or any central nervous system disease; pregnant or lactating women; and had undergone electroconvulsive therapy within the 3 months before the study.

### Study design and ethics approval

This study used a cross-sectional design. Potentially eligible participants were recruited through advertisements presenting in the outpatient/inpatient department. After screening the patients according to the inclusion and exclusion criteria by two senior psychiatrists, eligible patients and their guardians provided written informed consent. We collected their demographic information and conducted a clinical evaluation for eligible subjects on the same day of screening. All participating doctors received training and instructions on how to collect the required information. All data were recorded on the clinical record form during the evaluation. The lead investigator checked the clinical record form following its completion. If there were any omissions or errors, the accuracy of the information was checked and corrected accordingly. After all the clinical data were collected, EpiData version 3.1 was used to check for double data entry to ensure the accuracy of the entered data. All clinical record sheets and electronic databases were maintained and stored by the lead investigator and backed up in real-time to avoid data loss.

The study was approved by the Ethics Committee of PKU6H. All participants were informed of the study and provided written informed consent to participate. Participants aged under 18 years participated in the study with the consent of both their guardian and the participant.

### Assessment

#### Clinical features rating

The Negative Symptom Assessment (NSA) assesses the existence, severity, and scope of the negative symptoms of schizophrenia. Trained researchers conducted semi-structured interviews based on the circumstances of patients with schizophrenia during the previous week (25). The NSA was developed by Axelrod et al. and comprises 16 items (25). The Chinese version of the NSA (NSA-15) was adapted to a 15-item scale that excluded item 6 (Affect: Reduced modulation of intensity) and has been shown to have good reliability and validity in a Chinese population (21). The 15 items of the NSA-15 are scored on a six-point scale ranging from 1 (absent) to 6 (severe) and consists of three factors: communication, emotion, and motivation. The *communication factor* (items 1–4, 9, and 15–16) measures alogia, rapport with the interviewer, reduced gestures, and slow movement, depending on participants’ explicit performance on verbal or non-verbal reactions to questions during the interview. The *emotion factor* (items 5 and 7) measures the range of emotions and affect and evaluates participants’ appraisal and expression in the affective aspect. The *motivation factor* (items 8, 10–14) assesses social drive, sexual interest, hygiene, sense of purpose, range of interests, and daily activity and focuses on the initiative of social and interpersonal affairs (21). The test-retest and interrater reliability of the NSA-15 are comparable to or higher than those of other similar tools, such as the Brief Negative Symptoms Scale and the Clinical Assessment Interview for Negative Symptoms, which indicates that the NSA-15 is more reliable for measuring negative symptoms in Chinese patients (21). The correlation between the NSA-15 and other symptoms is relatively low, which signifies a boundary between negative symptoms and other symptoms (21). This helped us to precisely assess the relationship between negative symptoms and other symptoms by minimizing the overlap in measurement content.

The Positive and Negative Syndrome Scale (PANSS) is a widely used scale to evaluate the severity of schizophrenia symptoms, and the Chinese version has good reliability and validity (26). The scale is divided into three subscales: positive symptoms, negative symptoms, and general pathological symptoms. We used the PANSS five-factor model proposed by Jiang et al. (27), which comprises five factors: positive factors, which include P1 delusions, P3 hallucinations, P6 paranoia, and G9 abnormal thoughts; negative factors, which include N2 emotional withdrawal, N3 poor relationships, N4 passive social withdrawal, N6 lack of spontaneity in conversation, and G7 motor retardation; excitatory factors, which include P4 hyperactivity, P7 hostility, and G14 poor impulse control; depressive factors, which include G2 anxiety, G3 guilt, and G6 depression; cognitive factors, which include G10 disorientation and G12 lack of judgment and insight. In the present study, we measured positive symptoms, negative symptoms, depressive symptoms, excitement, cognitive symptoms using the corresponding factors of this PANSS model. PANSS total score was used to present the severity of the global psychotic symptoms.

The Calgary Depression Scale for Schizophrenia (CDSS) is used to assess the severity of depressive symptoms in patients with schizophrenia, and the Chinese version of the CDSS (CDSS-C) has been shown to be valid and reliable (28). The CDSS-C comprises nine items, and the cutoff score for the presence of depression is ≥6 points (29).

The Rating Scale for Extrapyramidal Side Effects (RSESE) was used to assess the extrapyramidal side effects of antipsychotic medications (30), and the Chinese version has been well-validated and shown to be reliable (31).

We adopted oral olanzapine equivalents based on defined daily doses to measure treatment intensity. The olanzapine equivalents were available for all the antipsychotics used by subjects and made the doses comparable (32).

#### Social functioning assessments

The Personal and Social Performance Scale (PSP) was designed to measure psychotic patients’ social functioning across four aspects: socially useful activities, personal and social relationships, self-care, and disturbing and aggressive behaviors (33). It was derived from the DSM-IV and Social and Occupational Functioning Assessment Scale (33). The Chinese version of the PSP has good reliability and validity (34). A total score of ≤30 points on the PSP indicates poor social functioning and the need for care and attention from others. A total score of ≥70 indicates good social functioning and the ability to take care of themselves and work. In this study, we measured social functioning and other clinical features of inpatient subjects based on their social performance of past month in the beginning week of hospitalization.

Employment status was reported by the participants and their guardians. We asked participants three questions: Are you currently a student? Have you worked for pay during the past 30 days? Have you done any casual work or day jobs? These questions were taken from the Services Utilization Recording Form developed by Rosenheck et al. (35). Participants who answered yes to any of the three questions were allocated to the employed/studying group, and those who gave negative answers were classified into the unemployed group.

### Statistical analysis

Data were analyzed using the Statistical Package for the Social Sciences software version 23 (SPSS, Chicago, IL, USA).

Continuous data and discrete numerical variables are presented as means and standard deviations. Categorical variables are described as frequencies (*n*) and percentages (%).

For the two types of social outcomes, the PSP total score was a continuous variable, and employment status was a categorical variable. Therefore, different statistical methods were used.

In the first stage, we analyzed the relationships among the three negative symptom dimensions and PSP score using correlation coefficients. The associations among the PSP score and potential confounding variables (i.e., age, sex, educational level, disease duration, and drug dose) and the other clinical characteristics were examined because numerous clinical features may impact social functioning. In the second stage, factors that were significantly associated (*p* < 0.05) in the first stage were included as independent variables in the stepwise linear multiple regression analysis to determine the cross-sectional determinants of the total PSP score.

For the association between employment and negative symptoms, demographic variables and clinical data were initially compared using one-way analyses of variance (ANOVAs) by employment status (i.e., employed/studying and unemployed groups). Then, logistic regression was performed to identify potential predictors of employment. The significance level was set at *p* < 0.05.

Multicollinearity is particularly important when a large number of factors are included in the linear regression model. In this study, a principle of variance inflation factor of ≤5 was used to construct the regression model to ensure the maximum practical significance of the model (20). To minimize multicollinearity and generate meaningful result, we excluded the total scores of PANSS and NSA from regression part. Also, PANSS-NS and PANSS-Dep were excluded since negative and depressive symptoms were represented by NSA and CDSS.

## Results

### Descriptive data

Demographic and clinical features are shown in Table 1. This study included 202 participants with schizophrenia (105 males and 97 females). The average age was 29.42 years old (SD: 10.00). The average duration of the disease was 8.12 years (SD: 7.38), and the shortest and the longest duration were 1 month and 38.5 years respectively.

**TABLE 1:** Demographics and clinical features.

	Mean (SD)
**Demographics**	
Sex	
Male (*n*/%)	105 (51.98%)
Female (*n*/%)	97 (48.02%)
Age (years)	29.42 (10.00)
Education (years)	13.48 (2.60)
Primary school (*n*/%)	5 (2.50%)
Secondary school (*n*/%)	88 (43.50%)
Higher vocational (*n*/%)	44 (21.80%)
University (*n*/%)	65 (32.20%)
Marital status	
Never married (*n*/%)	159 (78.70%)
Married/divorced (*n*/%)	43 (21.30%)
Hospitalization Inpatient Outpatient	141 (69.80%) 61 (30.20%)
**Clinical features**	
Age of psychosis onset	21.25 (7.02)
Illness duration	8.16 (7.38)
Dose of antipsychotics (equivalent to olanzapine)	17.16 (9.04)
PANSS	
PANSS total	57.64 (17.44)
PANSS-positive symptoms	9.89 (5.35)
PANSS-negative symptoms	11.70 (5.71)
PANSS-excitement	3.81 (1.69)
PANSS-depression	4.92 (2.47)
PANSS-cognition	5.06 (1.80)
NSA	
NSA total	41.18 (15.35)
NSA-communication	13.41 (6.29)
NSA-emotion	6.68 (2.73)
NSA-motivation	18.33 (7.00)
CDSS	
CDSS total	0.73 (1.99)
RSESE	
RSESE total	10.72 (1.43)
**Social functioning**	
Employment	
Studying/employed (*n*/%)	94 (46.5%)
Unemployed (*n*/%)	108 (53.5%)
PSP	
PSP total	53.33 (17.11)

PANSS, the positive and negative syndrome scale; PSP, the personal and social performance scale; NSA, negative symptom assessment; CDSS, the calgary depression scale for schizophrenia; RSESE, the rating scale for extrapyramidal side effects.

### Correlations between social functioning and clinical characteristics

The associations between social functioning and clinical characteristics are shown in Table 2. The PSP total score, representing global social functioning, was negatively correlated with the severity of symptoms. Specifically, the total PSP score was negatively correlated with the total NSA score (*r* = –0.694; *p* < 0.01). Among the three dimensions, NSA-Communication (*r* = –0.657; *p* < 0.001) and NSA-Motivation (*r* = –0.662; *p* < 0.001) were significantly negatively correlated with the PSP score, whereas the correlation between the PSP score and NSA-Emotion (*r* = –0.509; *p* < 0.001) was significant but weaker. The relationships between the PSP total score and PANSS total score (*r* = –0.680; *p* < 0.001) and the PANSS-Negative symptoms score (*r* = –0.686; *p* < 0.001) were similar. Other PANSS subdomain scores (PANSS-Positive symptoms, PANSS-Excitement, PANSS-Depression, and PANSS-Cognition scores) showed a weak yet significant negative correlation (*r*s = –0.356 –0.205; *p* < 0.005) with social functioning. However, depressive symptoms, represented by the CDSS total score, did not correlate with social functioning (*r* = –0.044; *p* = 0.536). The RSESE total score representing extrapyramidal side effects (*r* = –0.166; *p* = 0.018) showed a weak yet significant negative correlation with social functioning. The demographic variables of age (*r* = 0.213; *p* = 0.002) and educational years (*r* = 0.285; *p* < 0.001), hospitalization (r = –0.204; *p* = 0.004), antipsychotic dose (*r* = –0.164; *p* = 0.031), and extrapyramidal side effects (*r* = –0.166; *p* = 0.018) were included in the regression analysis as potential confounding variables because they were significantly related to social functioning.

**TABLE 2:** Correlations between variables.

	(coeffecient)	1	2	3	4	5	6	7	8	9	10	11	12	13	14	15	16	17	18	19	20	21	22
1	Sex	1																					
2	Marriage	0.180*	1																				
3	Employment	0.027	0.012	1																			
4	Age	0.099	0.528**	0.027	1																		
5	Years of edu	0.073	0.005	0.171*	0.198**	1																	
6	Age of onset	0.203*	0.368**	0.178	0.633**	0.251*	1																
7	Duration	0.076	0.327**	−0.182*	0.662**	0.024	–0.117	1															
8	Hospitalization	–0.015	−0.204**	–0.061	−0.166*	−0.236**	–0.157	−0.148*	1														
9	Dose	0.059	0.008	−0.186*	–0.063	–0.025	–0.184	0.043	0.194**	1													
10	NSA total	–0.053	–0.031	−0.172*	−0.226**	−0.320**	–0.037	−0.203**	0.205**	0.162*	1												
11	NSA-Com	–0.062	–0.038	–0.101	−0.250**	−0.272**	–0.031	−0.235**	0.179*	0.167*	0.893**	1											
12	NSA-Emo	–0.005	0.027	−0.140*	–0.131	−0.351**	–0.038	−0.147*	0.155**	0.081	0.798**	0.626**	1										
13	NSA-Moti	–0.037	–0.054	−0.224*	−0.194**	−0.254**	–0.025	−0.152*	0.189**	0.173*	0.925**	0.717**	0.664**	1									
14	PANSS total	0.006	0.019	`−0.080	–0.094	−0.154*	–0.083	−0.218**	0.151**	0.193*	0.665**	0.675**	0.479**	0.587**	1								
15	PANSS-PS	0.063	0.086	0.047	−0.221**	–0.019	0.079	−0.204**	0.083	0.238**	0.270**	0.319**	0.188**	0.207**	0.721**	1							
16	PANSS-NS	–0.053	–0.008	–0.091	–0.047	−0.203**	–0.076	−0.213**	0.131	0.073	0.860**	0.861**	0.591**	0.770**	0.765**	0.310**	1						
17	PANSS-Exc	0.066	0.010	′	0.006	0.014	–0.124	–0.020	–0.082	0.127	0.033	0.062	0.009	0.029	0.420**	0.351**	0.110	1					
18	PANSS-Dep	0.124	–0.020	–0.089	–0.099	0.067	0.046	–0.015	0.022	0.122	0.068	0.064	0.027	0.080	0.501**	0.399**	0.175*	0.356**	1				
19	PANSS-Cog	–0.003	–0.041	–0.110	−0.182**	–0.109	–0.169	−0.209**	0.245**	0.186*	0.290**	0.166*	0.354**	0.297**	0.426**	0.321**	0.234**	0.097	0.072	1			
20	CDSS total	0.137	0.025	–0.029	–0.057	0.031	`0.110	–0.062	–0.001	′	–0.030	–0.038	–0.047	0.006	0.230**	0.242**	0.038	0.076	0.569**	0.010	1		
21	RESES	0.071	–0.009	–0.074	–0.068	–0.108	–0.115	–0.019	0.055	0.123	0.293**	0.301**	0.251**	0.254**	0.161**	0.032	0.276**	–0.043	0.025	–0.086	–0.083	1	
22	PSP total	–0.024	0.087	0.215**	0.213**	0.285**	0.134	0.138	−0.204**	−0.164*	−0.694**	−0.657**	−0.509**	−0.662**	−0.680**	−0.356**	−0.686**	−0.205**	−0.241**	−0.288**	–0.044	−0.166*	1

Dose, dose of antipsychotics; NSA, negative symptom assessment; NSA-Com, NSA-communication factor; NSA-Emo, NSA-emotion factor; NSA-Moti, NSA-motivation factor; PANSS, the positive and negative syndrome scale; PANSS-PS, PANSS-positive symptoms factor; PANSS-NS, PANSS-negative symptoms factor; PANSS-Exc, PANSS-excitement factor; PANSS-Dep, PANSS-depression factor; PANSS-Cog, PANSS-cognitive factor; CDSS, the calgary depression scale for schizophrenia; RSESE, rating scale for extrapyramidal side effects; PSP, the personal and social performance scale. *p < 0.05; **P < 0.01.

Multiple linear regression with stepwise analysis (Table 3) revealed that the most significant predictor of social functioning was motivation, which had roughly three times the adjusted beta of positive symptoms or excitement (–0.472 vs. –0.134/–0.126, respectively) in model 4 and contributed 48.2% R^2^ independently in model 1. Model 2 was composed of two negative symptom dimensions (i.e., motivation and communication) and contributed 52.3% to the adjusted R^2^. Interestingly, the emotion factor of the NSA was not included in the regression model. Although depressive symptoms, cognitive symptoms, age, and antipsychotic dose showed significant correlations, they were excluded from the multiple regression model. According to R^2^, model 4, which included motivation, communication, positive symptoms, and excitement, explained 56.0% of the variance in social functioning. Compared with model 2, the two additional factors in model 4 (i.e., positive symptoms and excitement) only increased the R^2^ by 3.7%. Age, educational years, hospitalization, antipsychotic dose, and extrapyramidal side effects were excluded from the regression models with *p* > 0.05.

**TABLE 3:** Multiple stepwise regression analysis.

Model	Independent variables	Unadjusted *B* (95% CI)	β	*t*	*p*	Adjusted *R*^2^	VIF
1	NSA-motivation	−1.737 (−2.006, −1.468)	−0.697	−12.737	<0.001	0.482	1
2	NSA-motivation	−1.191 (−1.567, −0.815)	−0.478	−6.252	<0.001	0.523	2.117
	NSA-communication	−0.872 (−1.309, −0.436)	−0.301	−3.945	<0.001		2.117
3	NSA-motivation	−1.183 (−1.549, −0.817)	−0.475	−6.381	<0.001	0.548	2.117
	NSA-communication	−0.732 (−1.165, −0.299)	−0.253	−3.336	<0.005		2.202
	PANSS-positive symptoms	−0.546 (−0.877, −0.214)	−0.174	−3.246	<0.005		1.093
4	NSA-motivation	−1.177 (−1.538, −0.816)	−0.472	−6.433	<0.001	0.560	2.118
	NSA-communication	−0.726 (−1.153, −0.298)	−0.251	−3.351	<0.01		2.202
	PANSS-positive symptoms	−0.422 (−0.765, −0.079)	−0.134	−2.426	<0.05		1.202
	PANSS-excitement	−1.325 (−2.430, −0.219)	−0.126	−2.365	<0.05		1.117

PSP total score as dependent variables and social-psychological characteristics as predictors.

### Correlation between employment and clinical characteristics

Employment is considered a milestone of social functioning. The ANOVA for employment shows antipsychotic dose, NSA-Motivation, NSA-total score and hospitalization showed significant differences between the two groups (Table 4). NSA-Motivation (odds ratio [OR] = 0.925, 95% confidence interval [CI]: [0.881, 0.971]; *p* = 0.002) and hospitalization (OR = 2.316 CI: [1.149, 4.666]; *p* = 0.019) were the only two predictive factors (Nagelkerkerker *R*^2^ = 16.6%) (Table 5). NSA-total score was rejected for possible multicollinearity with NSA-Motivation.

**TABLE 4:** ANOVA for employment.

	*F*	*p*
**Employed/study group versus unemployed group**		
Sex	0.150	0.698
Age	0.972	0.325
Years of education	3.106	0.080
Hospitalization	10.444	0.001**
Age of psychosis onset	2.626	0.108
Illness duration	2.598	0.109
Dose of antipsychotics	5.633	0.019*
NSA-Com	1.286	0.258
NSA-Emo	3.191	0.076
NSA-Moti	10.533	0.001**
NSA-Total	5.697	0.018*
PANSS-PS	0.498	0.481
PANSS-NS	1.575	0.211
PANSS-Exc	0.182	0.670
PANSS-Dep	1.443	0.231
PANSS-Cog	2.643	0.106
PANSS-Total	1.141	0.287
CDSS	0.025	0.874
RSESE	0.001	0.985

Dose, dose of antipsychotics; NSA-Com, NSA-communication factor; NSA-Emo, NSA-emotion factor; NSA-Moti, NSA-motivation factor; PANSS-PS, PANSS-positive symptoms factor; PANSS-NS, PANSS-negative symptoms factor; PANSS-Exc, PANSS-excitement factor; PANSS-Dep, PANSS-depression factor; PANSS-Cog, PANSS-cognitive factor; CDSS, the calgary depression scale for schizophrenia; RSESE, rating scale for extrapyramidal side effects. PSP, the personal and social performance scale. *p < 0.05; **p < 0.01.

**TABLE 5:** Logistic regression analysis predicting employment status.

	Employed/study group versus unemployed group					
	*B*	SE	Wald	df	*P*	OR (95% CI)
NSA-Moti	–0.072	0.025	8.201	1	0.004**	0.931 (0.886, 0.978)
Dose of antipsychotics	–0.024	0.019	1.561	1	0.211	0.976 (0.940, 1.014)
Hospitalization	0.840	0.357	5.518	1	0.019*	2.316 (1.149, 4.666)

NSA-Moti, NSA-motivation factor.

*p < 0.05; **p < 0.01.

## Discussion

This study is the first to examine the role of a wide range of demographic and clinical characteristics in the relationship between dimensional negative symptoms and social functioning in Chinese patients with schizophrenia.

We found that the three dimensions of negative symptoms (i.e., emotion, motivation, and communication) were correlated with social functioning. Furthermore, motivation was the most significant predictor of social functioning, whereas communication showed an additional contribution. For vocational status, deficits in motivation were a significant predictor of unemployment, while communication or emotion failed to predict it. The results highlighted the significant relationship between motivation, instead of the other two negative symptoms domains, and social functioning.

We found that positive symptoms, cognitive symptoms, and excitement were all correlated with social functioning. Positive symptoms and excitement contributed to the prediction of social functioning, though the adjusted Betas showed that the predicting effects of them may be weaker than motivation or communication.

The effects related to medication may also impact social functioning. Extrapyramidal side effects were associated with all aspects of negative symptoms and social functioning. However, it was not related to employment and did not predict social functioning. The equivalent dose was found to be a possible, albeit non-significant, predictor of employment. Hospitalization also correlated with social functioning and predicted employment status.

We compared our findings with similar studies run in other regions. Our findings emphasized the association between avolition and social functioning, which is consistent with previous studies in Western and Asian populations (5, 15, 17, 20, 36). Rocca et al. investigated the association between social functioning and expressive deficits and avolition in a sample of Italian schizophrenia outpatients. They found that avolition is correlated with global social functioning, socially useful activities, personal and social relationships, and self-care (15), which were assessed by the PSP. Another study revealed that motivation is an exclusive predictor of functioning, accounting for 74% of the variance of current functioning, and also suggested that motivational deficits are the central link between negative symptoms and dysfunction in Canadian participants with schizophrenia (36). Ang et al. showed that motivation is correlated with an increased likelihood of unemployment in patients in Singapore, which is a developed Asian country made up by Chinese ethnic dominate (17). Okada et al. emphasized the association between residential outcome, entertainment and employment and avolition. They also stressed asociality, which is contained in the factor “motivation” in our study, related to withdrawal, social participation and interaction (20). The relationship between avolition and social functioning appears to be stable across different studies based on various populations.

Impairments in motivation, which are described by the terms *avolition* and *amotivation*, are characterized by reduced initiation and lack of persistence in goal-directed activities (5). They bring further difficulties in life and lead to poor social outcomes (5, 14). Avolition in schizophrenia is associated with poorer premorbid social adjustment in childhood, more insidious psychotic episodes, deficits in executive function and abstract flexibility, and male sex (5). Avolition is considered a unique dimension of the negative symptoms of schizophrenia because it is highly centralized and correlated with other negative symptom subdomains (37). Indeed, after successfully improving avolition, patients’ overall negative symptom clusters have also been shown to change significantly (37, 38). Therefore, it is important to highlight the importance of avolition as a negative symptom to develop more effective treatments targeted at improving patients’ social functioning (39).

However, because the mechanism underlying avolition is unclear, there has not been significant progress in treatments to improve motivation. Studies have shown that blunt dorsal striatal activity may be specifically associated with avolition (motivation) (40, 41). Additionally, avolition and anhedonia can be mapped to functional corticostriatal pathways (37). Although avolition may show regional specificity in the human brain, whether it is neuropathologically independent of other negative symptoms and how it interacts with other symptoms remain uncertain (19, 37). Strauss et al. suggested that the therapeutic effect is derived from the decreased centrality of avolition, which reduces the connectivity of other negative symptom domains (39). Several studies have indicated that to identify therapeutic targets for negative symptoms, studies may need to focus on the symptomology of different sexes (39). However, neither ours nor a previous study revealed significant sex differences in the subdomains of negative symptoms and social functioning (42). An impaired reward-predicting system and a decreased effort toward the pursuit of rewards may relate to avolition and offer therapeutic potential (19). There remain numerous contradictions in neuropathological and therapeutic research, and further research is needed to understand the pathophysiology of impaired motivation.

As for the possible intervention for motivation deficits, studies focused on both novel pharmacological and psychosocial treatments (43). Several clinical trials have demonstrated that treatments based on non-dopaminergic mechanisms are a promising future research direction because typical dopaminergic therapy is not effective in motivation improvement (38). For example, roluperidone, a compound with a high affinity for 5HT2A receptor and sigma-2 and alpha-1 adrenergic receptors, improves negative symptoms in terms of both emotional expression and experience (44). Some novel compounds focusing on NMDA glutamatergic system or cholinergic system also demonstrated potentials on treating negative symptoms and avolition (43). Psychosocial approaches also showed a potential efficacy on motivational deficits. Social skills training, as one of the conventional interventions for patients with social functional impairment, presented a small-to-medium improvements for negative symptoms, especially in the aspect of social motivation. Cognitive therapy for negative symptoms would help patients to address defeatist belief and encourage social interaction (43). We proposed that the future study could combine non-dopaminergic pharmacological treatment with psychological treatment together to enhance patients’ motivation and social functioning.

In previous studies on the dichotomous definition of negative symptoms, the impact of expression (including alogia and blunted affect) on social functioning has been shown to be controversial (45, 46), which may be attributed to the lack of key information due to the crudeness of the two-factor model (21, 46, 47). Huang et al. applied confirmatory analysis to identify a three-factor model that is suitable for the Chinese population (21). The expression (including alogia and blunted affect) factors in the two-factor model were regarded as the communication factor (i.e., alogia, lack of gesture, and slow movement) and the emotion factor (i.e., emotion and affect) in the three-factor model. Regression analysis showed that the communication factor, rather than the emotion factor, was a predictor of social functioning. Therefore, a possible reason for the inconsistent relationships reported between expression and social function is that only a specific element of expression (i.e., alogia, lack of gesture, and slow movement) is closely related to social functioning. Although alogia and blunted affect are strongly inter-correlated (48), it is clear that these two factors have different implications for social functioning, which may provide motivation for latent factor model analysis of negative symptoms to clarify the debate about the two-factor and three-factor models.

As for other factors related to social functioning, the results on positive symptoms were inconsistent with the findings of previous studies (48, 49). The discrepancy may appear because we recruited inpatients with an acute psychotic episode, whereas previous studies focused on stable patients with schizophrenia (i.e., those who had not been hospitalized in 6 months). Understandably, patients experiencing severe hallucinations or delusions leading to hospitalization would be expected to perform poorly. One study has suggested that social functioning is compromised by paranoia in the context of acute psychosis (50) and highlighted the impact of persistent positive symptoms on social functioning, which is consistent with our findings. Hospitalization also presented a significant association with social functioning and employment. However, due to the assessments on inpatient subjects happened on the first week of hospitalization, this correlation cannot extrapolate to patients with long-term hospitalization.

This study identified possible influencing factors on the social functioning of patients with schizophrenia. We applied a three-factor model that was suitable for the characteristics of the participants, which made our conclusions more credible. Our study sample was relatively representative, with disease duration ranging from 1 month to 39 years. Additionally, we considered various potential confounding factors, including demographics, medication status, and extrapyramidal side effects, to minimize the confounding effects.

This study has several limitations. First, because we used a cross-sectional design, we were unable to examine the relationship between the variation of each factor over time. Further cohort studies are needed to clarify the relationship between negative symptom dimensions and social functioning and the underlying causes. Second, although we included numerous clinical features, social cognitive and neural cognitive test data were lacking, which may have created a gap in the predictive model. Third, we recruited participants in a real-world setting, including inpatients, outpatients, and patients in different phases of an episode, to ensure the representativeness of the sample. However, the sample size was still limited. Larger samples should be expected in future studies to confirm the association between negative symptoms and social functioning. Additionally, we suggest that future studies conduct subgroup analyses by hospitalization or phases of episodes to gain a more detailed understanding of the mechanism underlying the association.

## Conclusion

Social functioning in schizophrenia was significantly related to motivation. We propose that future studies focus on deficits in motivation and consider it a therapeutic target to improve social functioning.

## Data availability statement

The raw data supporting the conclusions of this article will be made available by the authors, without undue reservation.

## Ethics statement

The studies involving human participants were reviewed and approved by Ethics Committee of Peking University Sixth Hospital. The adult participants provided their written informed consent to participant in this study. The participants aged under 18 participated in the study with the consent of both their guardian and the participant.

## Author contributions

XY, CP, and CS designed the study, obtained funding, and supervised the study. TG, CP, and XY performed the data analysis and interpretation. TG, CP, and XY conducted the research design and manuscript writing. ZH, BH, TZ, and CS participated in the collection of data. All authors contributed to the article and approved the final version for publication.
